# Patient-Reported Outcomes After Same-Day Mastectomy Among Older Breast Cancer Patients: Results From a Prospective Clinical Trial

**DOI:** 10.1155/tbj/9953747

**Published:** 2025-11-07

**Authors:** Jessica C. Gooch, Qi Ying McClelland, Kathryn Paschalis, Maya Anand, Allison Magnuson, Jenna Dobbins, Kristin A. Skinner, Ann Olzinski-Kunze, Anna Weiss

**Affiliations:** ^1^Division of Surgical Oncology, Department of Surgery, University of Rochester Medical Center, Rochester, New York, USA; ^2^Wilmot Cancer Institute, University of Rochester Medical Center, Rochester, New York, USA; ^3^Department of Surgery, University of Rochester Medical Center, Rochester, New York, USA; ^4^University of Rochester School of Medicine and Dentistry, Rochester, New York, USA; ^5^Division of Medical Oncology, Department of Medicine, University of Rochester Medical Center, Rochester, New York, USA

**Keywords:** breast cancer, elderly, enhanced recovery after surgery, patient-reported outcomes, same-day discharge, same-day mastectomy

## Abstract

**Background:**

The safety and value of same-day mastectomy are well-documented but the patient perspective is underreported, especially among older patients. This study aimed to investigate older patient-reported recovery quality after mastectomy; we hypothesized that patients who were discharged same day would report better recovery.

**Methods:**

A prospective trial included frailty screening and prehabilitation for patients age ≥ 65 undergoing mastectomy for breast cancer. Primary endpoint, same-day discharge rate, was previously reported and was significantly higher than the year prior. Secondary endpoint was patient-reported postoperative recovery quality, per the Quality of Recovery-15 measure (QoR-15; 15 questions scored 1–10, 10 being best). Patients responded by phone 24–72 h postdischarge. One-tailed *T*-tests compared responses between same-day and admitted patients.

**Results:**

37/55 (67.3%) patients ≥ 65 who underwent unilateral/bilateral mastectomy for early-stage breast cancer responded. Mean age was 73.6 (standard deviation 7.6), most had invasive carcinoma (44, 80.0%), and mean 5-factor Modified Frailty Index (mFI-5) was 1.3 of 5 (standard deviation 0.9); nonresponders had similar characteristics. There were no significant differences in any QoR-15 item (all *p* > 0.05). In fact, most responses were very similar, different by only one-tenth of 1 point or identical. The following answers slightly (0.2 difference or more) numerically favored same-day discharge: feeling rested, having good sleep, less moderate pain, and freedom from feeling anxious or depressed. No items favored admission.

**Conclusions:**

Although this trial was not powered for secondary analyses, it is clinically meaningful that older patients undergoing same-day mastectomy reported similar recovery quality as those admitted. Same-day mastectomy should be considered for older patients.

## 1. Introduction

The safety [1–3] and value [4] of same-day mastectomy are well-documented. However, the mean age of patients studied is relatively young, about 50 years in some reports [2]. In a large integrated healthcare system, only about 1/3 of patients were aged ≥ 65 years [5]. It is logical that surgeons tend to admit older patients, as older age and increasing numbers of comorbid conditions are known risk factors for postoperative complications [6, 7]. However, mastectomy is a minimal risk surgery, and admission of elderly patients based on age alone is anecdote.

Our institution has a strong geriatric oncology program, and its providers felt that discharging elderly patients would be best for them as they may experience less delirium, less falls, and be more comfortable recuperating in their own home. Thus, we started a prospective protocol to assess patients aged ≥ 65 years for frailty, via the 5-factor Modified Frailty Index (mFI-5), refer to geriatric oncology for prehabilitation if frail, and encourage same-day discharge of all older patients as appropriate. The primary endpoint for the protocol, same-day discharge rate, was previously reported and was significantly higher than the year prior. Between March 2022 and February 2023, 18.8% of patients ≥ 65 years of age were discharged the same day as their mastectomy and then after protocol implementation, 56.1% were discharged the same day (*p* < 0.00001) [1].

Despite ample safety data, the patient perspective following same-day mastectomy is woefully underreported [8]. In fact, to our knowledge, there is no patient-reported outcomes data among older patients after same-day mastectomy. To that end, this study aimed to investigate patient-reported recovery quality after mastectomy, specifically among patients ≥ 65 years of age enrolled to our institutional prospective protocol; we hypothesized patients who were discharged same-day would report better recovery.

## 2. Methods

### 2.1. Patients

Patients ≥ 65 years of age were screened prospectively from the institution's surgeon schedules via electronic medical record review. For each patient, the 5-Factor mFI was calculated, and if the score indicated moderate-high frailty, a referral to geriatric oncology for prehabilitation was encouraged. The patients and their surgeons made operative decisions per standard care. Subsequent data from the patients who underwent mastectomy were prospectively collected; these patients comprised our study cohort.

### 2.2. Endpoints

The prospective protocol's primary endpoint, same-day discharge rate, was previously reported and was significantly higher than the year prior [1]. The sample size needed to see a meaningful increase in the percentage of patients discharged the same-day was determined to be 55 patients over the course of 1 year, based on the number of patients ≥ 65 years of age who underwent mastectomy and the number of patients who were discharged the same-day in fiscal year 2022. This meaningful increase would result in greater than 50% of patients discharged per key stakeholders including attending and resident surgeons, geriatric oncologists, and patient advocates. The secondary endpoint was patient-reported postoperative recovery quality, per the Quality of Recovery-15 measure (QoR-15; 15 questions scored 1–10, 10 being best). The QoR-15 measure, which includes 15 questions, was adapted from the QoR-40 which had 40 questions and validated [9]. Each question is rated on a scale 0–10. For the first 10 questions, 0 means “none of the time” and 10 means “all of the time.” For the last 5 questions (Moderate pain, Severe pain, Nausea or vomiting, Feeling worried or anxious, and Feeling sad or depressed), it is the reverse—10 means “none of the time” and 0 means “all of the time.” Thus, for all questions, a higher score represents better recovery. Patients were contacted by phone 24–72 h postdischarge to answer the survey questions. If no response, they were called weekly 2 additional times, for a total of 3 contacts.

### 2.3. Data Analysis

Categorical variables, presented as *n* (percentage), and continuous variables, presented as mean (standard deviation), were compared using chi square and two-tailed *T*-tests, respectively. We hypothesized that patients who were discharged home would report higher QoR scores, favoring one group, so one-tailed *T*-tests were utilized to compare QoR responses between same-day and admitted patients.

## 3. Results

### 3.1. Patient Characteristics

There were 55 total patients ≥ 65 years of age who underwent unilateral/bilateral mastectomy for early-stage breast cancer who comprised our study cohort. Two of these patients had bilateral disease. Mean age was 73.6 (standard deviation 7.6), most had invasive carcinoma (44, 80.0%) as opposed to in situ, and their mean 5-factor mFI score was 1.3 of 5 (standard deviation 0.9). Thirty (54.5%) patients were discharged on the same day as their mastectomy and 25 (45.5%) were admitted.  Table 1 presents the characteristics of patients who were discharged on the same day versus those who were admitted, with the length of stay ≥ 1 day. The only significant difference between patient groups was that more same-day discharge patients were treated with neoadjuvant therapy (10/30 [33.3%]) as compared to patients with the length of stay ≥ 1 day (2/25 [8.0%], *p*=0.02). There were no significant differences in discharge rates between different surgeons (*p*=0.215).

### 3.2. QoR-15 Results

**Figure 1:**
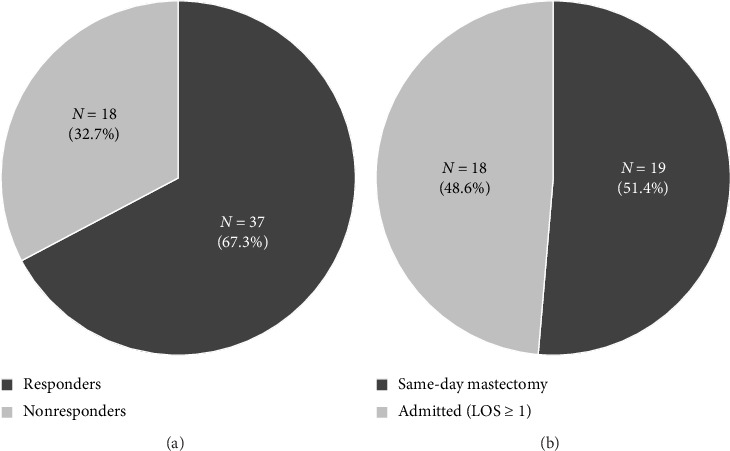
Overall response rate (a) and percent of responders who were discharged the same day or admitted with lengths of stays of 1 day or greater (b).

Thirty-seven of the 55 (67.3%) patients responded (Figure 1). Responders and nonresponders had similar patient and tumor characteristics (Table 2). Of the 37 responders, 19 (51.4%) were discharged the same day after their mastectomy and 18 (48.6%) were admitted; patient and tumor characteristics among responders who were or were not discharged were also similar. There were no significant differences in any QoR-15 item response between patients who were discharged immediately following their mastectomy or those who were admitted (all *p* > 0.05) (Table 3). In fact, most responses were very similar being different by only one-tenth of 1 point or identical. The following answers slightly (0.2 difference or more) numerically favored same-day discharge: feeling rested, having good sleep, less moderate pain, and freedom from feeling anxious or depressed. No items favored admission.

### 3.3. QoR-15 Results Among Different Patient Subsets

**Figure 2:**
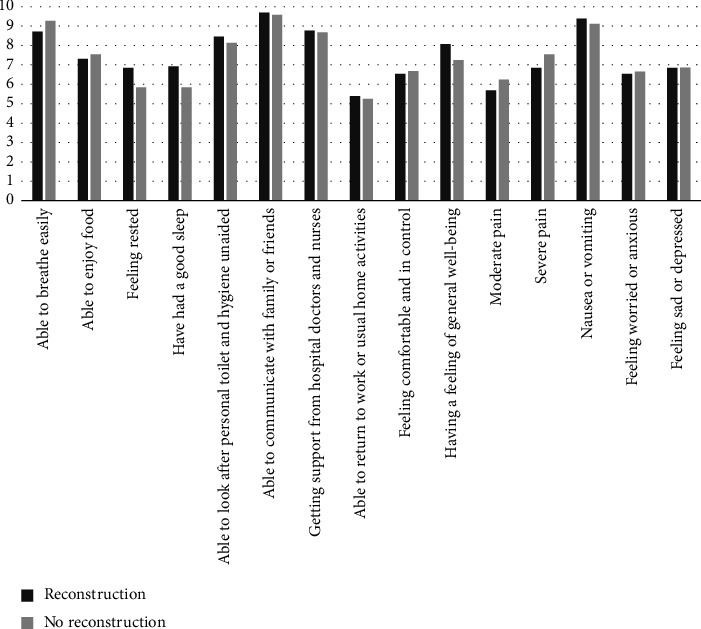
QoR-15 scores for patients who underwent reconstruction versus those who did not. Scores range from 0 to 10.

Five patients underwent immediate reconstruction, all with tissue expanders; 4 of these patients were survey responders, 2 who were discharged the same-day and 2 who were admitted. The following numerically favored reconstruction: feeling rested, having good sleep, being able to look after personal toilet and hygiene unaided, ability to return to work or usual home activities, having a feeling of general well-being, and freedom from nausea or vomiting (Figure 2). The following numerically favored no reconstruction: ability to breathe easily, ability to enjoy food, feeling comfortable and in control, and less moderate and severe pain.

**Figure 3:**
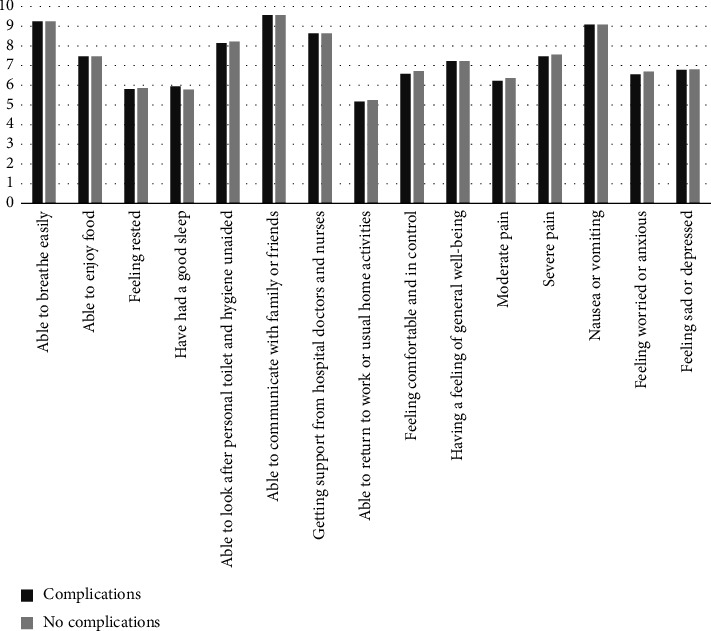
QoR-15 scores for patients with complications versus those without. Scores range from 0 to 10.

Sixteen of 55 patients (29.0%) had at least 1 complication. Among the 30 patients who were discharged the same day, 7 patients had 9 complication events including 1 hematoma, 2 infections, 3 with drain issues, 2 emergency room (ER) visits, and 1 call for pain medications. Among the 25 who were admitted, 9 patients had a total of 21 complication events, including 5 hematomas, 4 infections, 1 with drain issues, 5 readmissions, 4 ER visits, and 2 calls for pain medications. Eleven of the 16 patients with any complication were also survey responders. QoR among patients with complications were nearly identical to those without complications (Figure 3).

## 4. Discussion

In a prospective trial of same-day discharge after mastectomy for breast cancer, 30 (54.5%) of 55 patients ≥ 65 years of age were successfully discharged. Thirty-seven of the 55 patients (67.3%) included in the prospective protocol responded to the QoR-15 survey, 19 of which were discharged the same day and 18 of which were admitted. There were no significant differences in the QoR between elderly patients who were discharged immediately versus admitted after their mastectomy. This supports the same-day discharge of elderly patients after mastectomy.

Patient characteristics were similar between those who were discharged after their mastectomy versus admitted, except that there were significantly more patients treated with neoadjuvant therapy among the same-day mastectomy group. There was no apparent increase in complication rates after mastectomy among patients treated with neoadjuvant chemotherapy [10], indicating that same-day discharge remains a safe and data-driven approach even among patients who providers might reflexively assume would be higher risk. There were no differences between patients who did or did not respond to the survey, and no differences among the respondents whether they were discharged same day or admitted after mastectomy.

Patients who were discharged immediately after mastectomy reported a similar, nearly identical, QoR as those who were admitted. Responses to the following domains numerically favored same-day discharge by a very small amount: feeling rested, having good sleep, less moderate pain, and freedom from feeling anxious or depressed. There was no QoR mean response that favored admission. This is similar to the minimal other published patient-reported outcomes data for patients who were discharged same-day after mastectomy. In a study examining same-day discharge after mastectomy and immediate breast reconstruction, similar percentages of patients wished they had stayed in the hospital longer, felt ready for discharge, had pain control at night, needed additional postoperative appointments, and needed narcotics past 1 week [8]. With this, our current study supports the immediate discharge of patients following mastectomy, even if they are of an older age.

Only 5 patients underwent immediate reconstruction in this study, so our sample size is too small to confirm meaningful differences. Interestingly, feeling rested, getting good sleep, and freedom from nausea favored reconstruction. On the other hand, it was not surprising that the ability to breathe easily and freedom from moderate–severe pain favored no reconstruction. More work should be done to collect recovery information following reconstruction from elderly patients, which may be challenging given the relative paucity of older adults who choose to pursue reconstruction. This study provides modest evidence that elderly patients may be discharged after mastectomy, with or without reconstruction.

Interestingly, there were no discernible differences in QoR-15 scores among patients with complications versus not. As complications tend to occur several days to weeks after surgery, it is not surprising that the groups are similar as the survey questions are intended to rate the quality of the recovery in the immediate postoperative period. Longer-term patient-reported outcomes data should be studied among patients who suffer from operative complications after breast surgery.

Though this pilot study is interesting and novel, there are limitations. Our most significant challenge is the small sample size, which may have prevented the detection of significant differences in QoR-15 responses among discharged versus admitted patients or may have led to falsely differentiated QoR-15 findings among reconstruction patients. Second, our survey responders were not diverse—100% were of non-Hispanic white ethnicity and race. This is likely due to there being a small number of diverse patients to begin with, as opposed to response bias. All patients were approached in the exact same way—with the exact same method of contact and with the exact same number of follow-ups. Though this is a small sample size overall so conclusions are difficult to make, the lack of diverse patients may have skewed our results further and limited its generalizability. Future work may seek to incorporate more in-depth patient-reported outcomes and qualitative responses from patients about their experiences in the perioperative period.

Despite its weaknesses, this study is impactful. It lends additional support to discharging patients, regardless of age, immediately after mastectomy. Successful discharge of mastectomy patients requires institutional support, provider consensus, and patient education. In a follow-up study of a large integrated healthcare system discharging patients the same day after their mastectomy, investigators found that surgeon volume and multimodal pain medication administration increased the odds of successful home recovery [11]. The American Society of Breast Surgeons endorses same-day discharge after mastectomy, noting that pre-emptive management of nausea and pain is important for successful discharge [3]. In the current study, nausea and pain were not a major issue for patients; rather, patients reported the poorest recovery quality for the ability to get good sleep, feel rested, and return to usual activities. The current study provides insight into postoperative issues specific to elderly patients, and sleep disturbances should be further investigated to improve postoperative recovery for patients over 65.

Lastly, provider and/or selection bias needs to be addressed to successfully discharge elderly patients immediately following mastectomy. In a qualitative study, surgeons identified several barriers to successful discharge including patient knowledge, surgeon awareness of the evidence, anecdote, and colleague influence [12]. The best ways to address these barriers include institutional support to establish recovery protocols and provide patient education and to continue to produce evidence like this to provide surgeons with data upon which they can make evidence-based change to their practices.

## 5. Conclusions

In a prospective protocol, patients ≥ 65 years of age undergoing mastectomy were screened for frailty and sent for prehabilitation as appropriate, and discharge home was encouraged. Although this prospective trial was not powered for secondary analyses, it is clinically meaningful that older patients undergoing same-day mastectomy reported similar recovery quality as those admitted. Same-day mastectomy should be considered for older patients.

## Figures and Tables

**Table 1:** Patient characteristics, by length of stay.

Variable	Same-day discharge patients, *n* (%)	LOS ≥ 1 patients, *n* (%)	*p* value
Total, *n* (%)	30 (54.5%)	25 (45.5%)	—
Age (mean years; standard deviation)	72.5; 5.5	74.9; 9.4	0.25
Race			0.85
White	28 (93.3%)	23 (92.0%)	
Black	2 (6.7%)	2 (8.0%)	
Ethnicity			0.90
Hispanic/Latinx	1 (3.3%)	1 (4.0%)	
Non-Hispanic	29 (96.7%)	24 (96.0%)	
Frailty scores (mean score; standard deviation)	1.2; 0.9	1.4; 1.0	0.29
Histology			0.50
Invasive	23 (76.7%)	21 (84.0%)	
In situ (DCIS or Paget's)	7 (23.3%)	4 (16.0%)	
Estrogen receptor status			0.62
Positive	25 (83.3%)	22 (88.0%)	
Negative	5 (16.7%)	3 (12.0%)	
Progesterone receptor status			0.87
Positive	21 (70.0%)	17 (68.0%)	
Negative	9 (30.0%)	8 (32.0%)	
HER2 receptor status			0.20
Positive	1 (3.3%)	1 (4.0%)	
Negative	21 (70.0%)	22 (88.0%)	
Unknown or equivocal	8 (26.7%)	2 (8.0%)	
Pathologic tumor size (mean cm; standard deviation)	3.3; 3.1	3.8; 3.4	0.66
Pathologic N stage (p or yp)			0.27
Unknown	7 (23.3%)	2 (8.0%)	
N0	18 (60.0%)	16 (64.0%)	
N1	2 (6.7%)	5 (20.0%)	
N2-N3	3 (10.0%)	2 (8.0%)	
Neoadjuvant therapy			0.02^∗^
Yes	10 (33.3%)	2 (8.0%)	
Chemotherapy	5 (16.7%)	2 (8.0%)	
Hormonal therapy	5 (16.7%)	0 (0.0%)	
No	20 (66.7%)	23 (92.0%)	

*Note:* Bold indicates statistical significance.

Abbreviation: DCIS, ductal carcinoma in situ.

^∗^This *p* value compared neoadjuvant therapy yes versus no.

**Table 2:** Patient characteristics, among those who responded to the QoR-15 survey (survey responders) versus those who did not respond (survey nonresponders).

Variable	Survey responders, *n* (%)	Survey nonresponders, *n* (%)	*p* value
Total, *n* (%)	37 (67.3%)	18 (32.7%)	—
Age (mean years; standard deviation)	73.4; 7.0	74.1; 8.7	0.74
Race			—
White	37 (100.0%)	14 (77.8%)	
Black	0 (0.0%)	4 (22.2%)	
Ethnicity			—
Hispanic/Latinx	0 (0.0%)	2 (11.1%)	
Non-Hispanic	37 (100.0%)	16 (88.9%)	
Frailty scores (mean score; standard deviation)	1.3; 0.8	1.2; 1.2	0.71
Histology			0.31
Invasive	31 (83.8%)	13 (72.2%)	
In situ (DCIS or Paget's)	6 (16.2%)	5 (27.8%)	
Estrogen receptor status			0.86
Positive	33 (89.2%)	14 (77.8%)	
Negative	4 (10.8%)	4 (22.2%)	
Progesterone receptor status			0.77
Positive	26 (70.3%)	12 (66.7%)	
Negative	11 (29.7%)	6 (33.3%)	
HER2 receptor status			0.73
Positive	1 (2.7%)	1 (5.6%)	
Negative	30 (81.1%)	13 (72.2%)	
Unknown or equivocal	6 (16.2%)	4 (22.2%)	
Pathologic tumor size (mean cm; standard deviation)	3.1; 2.8	4.3; 4.0	0.19
Pathologic *N* stage (p or yp)			0.40
Unknown	7 (18.9%)	2 (11.1%)	
N0	21 (56.8%)	13 (72.2%)	
N1	7 (18.9%)	1 (5.6%)	
N2-N3	2 (5.4%)	2 (11.1%)	
Neoadjuvant therapy			0.15^∗^
Yes	6 (16.2%)	6 (33.3%)	
Chemotherapy	3 (8.1%)	4 (22.2%)	
Hormonal therapy	3 (8.1%)	2 (11.1%)	
No	31 (83.8%)	12 (66.7%)	
Length of stay (mean days; standard deviation)	0.5; 0.6	1.7; 4.9	0.17

Abbreviation: DCIS, ductal carcinoma in situ.

^∗^This *p* value compared neoadjuvant therapy yes versus no.

**Table 3:** Quality of recovery-15 responses, by length of stay.

Domain (mean score, standard deviation)	Same-day discharge patients, *n* (%)	LOS ≥ 1 patients, *n* (%)	*p* value
Total, *n* (%)	19 (51.4)	18 (48.6)	—
Able to breathe easily	9.2; 1.4	9.3; 1.3	0.17
Able to enjoy food	7.4; 3.2	7.5; 3.1	0.47
Feeling rested	6.1; 3.0	5.8; 3.0	0.37
Have had a good sleep	6.0; 3.3	5.8; 3.4	0.42
Able to look after personal toilet and hygiene unaided	8.1; 2.8	8.1; 2.7	0.19
Able to communicate with family or friends	9.6; 0.9	9.6; 0.9	0.47
Getting support from hospital doctors and nurses	8.6; 2.4	8.7; 2.3	0.33
Able to return to work or usual home activities	5.1; 3.5	5.2; 3.5	0.17
Feeling comfortable and in control	6.6; 3.1	6.7; 3.0	0.17
Having a feeling of general well-being	7.3; 2.6	7.2; 2.5	0.23
Moderate pain	6.4; 3.4	6.2; 3.5	0.10
Severe pain	7.5; 3.4	7.5; 3.3	0.11
Nausea or vomiting	9.2; 2.3	9.1; 2.4	0.17
Feeling worried or anxious	6.8; 3.3	6.6; 3.4	0.24
Feeling sad or depressed	7.0; 3.3	6.9; 3.4	0.21

## Data Availability

The data that support the findings of this study are available from the corresponding author upon reasonable request.

## Nomenclature

QoR: Quality of recovery
mFI: Modified Frailty Index
ER: Emergency room
